# Pediatric Headache Clinic Model: Implementation of Integrative Therapies in Practice

**DOI:** 10.3390/children5060074

**Published:** 2018-06-12

**Authors:** Anna Esparham, Anne Herbert, Emily Pierzchalski, Catherine Tran, Jennifer Dilts, Madeline Boorigie, Tammie Wingert, Mark Connelly, Jennifer Bickel

**Affiliations:** Children’s Mercy Hospital, University of Missouri-Kansas City School of Medicine, Division of Child Neurology, 2401 Gillham Rd., Kansas City, MO 64108, USA; ajherbert@cmh.edu (A.H.); ecpierzchalski@cmh.edu (E.P.); ctq44@mail.umkc.edu (C.T.); jjdilts@cmh.edu (J.D.); meboorigie@cmh.edu (M.B.); twingert@cmh.edu (T.W.); mconnelly1@cmh.edu (M.C.); jlbickel@cmh.edu (J.B.)

**Keywords:** headache, migraine, integrative medicine, complementary therapies, pediatric, mind-body, clinic model, multidisciplinary, implementation

## Abstract

The demand for integrative medicine has risen in recent years as research has demonstrated the efficacy of such treatments. The public has also become more conscientious of the potential limitations of conventional treatment alone. Because primary headache syndromes are often the culmination of genetics, lifestyle, stress, trauma, and environmental factors, they are best treated with therapies that are equally multifaceted. The Children’s Mercy Hospital, Kansas City, Missouri Headache Clinic has successfully incorporated integrative therapies including nutraceuticals, acupuncture, aromatherapy, biofeedback, relaxation training, hypnosis, psychology services, and lifestyle recommendations for headache management. This paper provides a detailed review of the implementation of integrative therapies for headache treatment and discusses examples through case studies. It can serve as a model for other specialty settings intending to incorporate all evidenced-based practices, whether complementary or conventional.

## 1. Introduction

Headaches are one of the most common disorders in childhood, with 60–75% of children reporting that they have had a significant headache by the age of 15 [1,2]. Headache disorders in children are truly a biopsychosocial phenomenon, best addressed with a holistic approach utilizing multidisciplinary therapeutic strategies [3,4]. Integrative medicine aims to make use of all evidence-based therapies, whether complementary or conventional, especially when treating chronic conditions [5,6]. Integrative medicine fosters optimal team dynamics, supports interdisciplinary providers and their staff, encourages healthy communication and mutual respect, and ultimately results in better care for patients [5]. As certain integrative therapies have been proven effective for certain conditions, healthcare providers may benefit from an understanding of the “how-to” of practical implementation of these integrative therapies into their practice [3,4].

Several studies have demonstrated that integrative therapies such as acupuncture, biofeedback, relaxation and stress coping skills, nutraceuticals/supplements, and aromatherapy are efficacious for headaches [7,8,9,10,11,12,13,14]. The use of complementary and integrative therapies in children with headaches may be as high as 76% in some populations [15]. Non-pharmacologic therapies implemented in practice with conventional medicine may prevent medication overuse and chronification of headaches. Because medical prophylaxis alone may often be insufficient, integrative approaches optimize clinical outcomes and provide patients with the best treatment experience [3,16]. Integrative therapies also increase patient self-care and empowerment by taking steps toward better health [17].

Much of the literature on integrative and complementary treatment modalities focuses on the public’s use of such therapies as well as their efficacy and safety. There is a paucity of literature on the various ways in which integrative therapies can be implemented in clinical practice. We describe how integrative therapies have been incorporated into a pediatric headache clinic in a Midwest academic medical center, Children’s Mercy Hospital and Clinics, Kansas City, Missouri (CMH). In this descriptive article, we outline integrative therapies and the evidence to support their use. Utilizing case studies, we describe how integrative therapies have been successfully incorporated into an academic headache practice. The case studies were deemed exempt by the CMH institutional review board.

## 2. Integrative Approaches and Their Implementation in Practice

### 2.1. Nutraceuticals/Dietary Supplements

Magnesium, riboflavin, and coenzyme Q10 are dietary supplements that are more commonly used for migraine headaches, based on the American Academy of Neurology and American Headache Society (AHS/AAN) guidelines [7]. Magnesium is involved in over 300 enzymatic reactions and participates in neuronal homeostasis through antagonizing the *N*-methyl-d-aspartate (NMDA) receptor, blocking calcium channels, and decreasing neurogenic inflammation [18]. Riboflavin plays a role in membrane stability, energy-related cellular function, and mediation of oxidative stress. Coenzyme Q10 (CoQ10) is involved in healthy mitochondrial function and acts as an antioxidant [19]. Butterbur exhibits anti-inflammatory effects by inhibiting leukotriene synthesis and regulating calcium channels [20]. Magnesium and riboflavin are considered by the AHS and AAN as Level B evidence: Probably effective for migraine prevention [7]. CoQ10 is considered Level C: Possibly effective for migraine prevention [7]. The American Academy of Neurology withdrew butterbur from their 2012 migraine prevention guidelines due to the associated risk of hepatotoxicity, as butterbur may contain petazolides [21]. The dietary supplement industry does not regulate the removal of petazolides, so caution is required.

In the CMH pediatric headache clinic, providers typically initiate a preventive supplement and/or medication for patients having four or more headaches per month. Preventive supplements or medications are each trialed one at a time for three months in order to determine therapeutic effect with the goal of decreasing headache severity and frequency. Potential risks, benefits, and side effects of supplements and medications are discussed with patients and their families to determine which preventive option is the best fit.

### 2.2. Acupuncture

Acupuncture’s mechanisms of action include activating the endorphin-enkephalin system, inhibiting pain pathways in the spinal cord and midbrain through neurotransmitter release, and cell-to-cell signaling through connective tissue manipulation [22,23,24,25]. Acupuncture has demonstrated efficacy for prevention of both episodic migraines and episodic tension-type headaches [8,9]. A 2016 Cochrane review demonstrated that acupuncture may be more effective at reducing migraine frequency compared to prophylactic drug treatment (Standard Mean Deviation (SMD) −0.25; 95% Confidence Interval (CI) −0.39–−0.10: 3 trials, 739 participants), but not at long-term follow-up [8]. Migraine frequency decreased by 50% in 41% of participants receiving acupuncture versus 17% of those not receiving acupuncture (Risk Ratio (RR) 2.40; 95% CI 2.08–2.76; 4 studies; 2519 participants) [8].

One large trial of 1265 participants in a Cochrane review, comparing acupuncture to routine care in the treatment of tension type headaches, showed a 50% reduction of headache frequency in 48% of acupuncture recipients versus 19% of those with routine treatment (RR 2.52; 95% CI 2.11–3.02; number needed to treat 3; 95% CI 3–5) [9]. A second trial of 207 participants, comparing acupuncture to routine care found 45% versus 4% improvement, respectively; (RR 11.36; 95% CI 3.69–34.98; number needed to treat 2; 95% CI 1–9) [9].

Acupuncture began to be utilized for our headache patients in 2015. Dr. Jennifer Bickel, MD, a neurologist and medical acupuncturist, developed the acupuncture program, which now includes three medical acupuncturists, all trained via a 300 h medical acupuncture course. According to a market data report by the CMH de-identified information database approved by CMH, the majority of patients treated with acupuncture included older adolescents, predominantly female, and of white race. One hundred and thirty patients received acupuncture between October 2015 and November 2017. The average number of acupuncture visits per patient was 4.1. The number of new and total acupuncture visits increased in relation to the addition of medical acupuncturists. Patients with refractory headaches were more likely to receive acupuncture, compared to patients with less frequent headaches. A significant proportion of acupuncture patients had also tried more aggressive treatments, such as OnabotulinumtoxinA injections (16%) and valproic acid (10%), suggesting that the patients receiving acupuncture had complex and intractable headaches. 

CMH marketed the acupuncture program by developing a page on their website that launched in April 2017. The acupuncture page has received nearly 200 views and is currently ranked second in Google searches for the keyword phrase “acupuncture for headaches in children”. The acupuncture clinic has expanded to include referrals from other CMH providers, predominantly from general neurology, pain management, rehabilitation, gastroenterology, and physical therapy. Acupuncture training privileges for acute pain protocols have also recently been approved by the CMH credentialing committee. The acupuncture training program will target physicians in the acute care setting (i.e., emergency department, urgent care) teaching two acute pain protocols.

### 2.3. Aromatherapy

Aromatherapy refers to the medicinal or therapeutic use of essential oils derived from plant sources [26]. Lavender essential oil has been commonly used for its anxiolytic and mood stabilizing properties. It has been evaluated for migraines in a placebo-controlled clinical trial. Inhaling lavender essential oil for 15 min reduced headache severity on the Visual Analogue Scale (VAS) from 3.6 ± 2.8 to 1.6 ± 1.6 and was statistically significant compared to the control group [10].

Aromatherapy started in the CMH Headache Clinic as a nurse-driven project, utilized as an adjunctive therapy with acupuncture. CMH provided training to personnel on aromatherapy through an online module course. Acupuncture patients were offered a hospital-approved lavender essential oil patch and asked to evaluate their experience. The survey asked if “using the essential oil patch enhanced my acupuncture experience” on a Likert scale of 1 (not at all) to 5 (very much). Twelve respondents completed the survey with an average Likert scale score of 3.5. All respondents unanimously responded “yes” to “I would like to use essential oil patches in the future”. On the basis of the positive responses, lavender essential oil patches continue to be utilized during acupuncture. 

### 2.4. Evidence-Based Behavioral and Self-Regulation Interventions

The United States Headache Consortium Guidelines has recommended (grade A evidence) that relaxation, biofeedback, and cognitive behavioral therapies may be considered as options for the prevention of migraines [27].

#### 2.4.1. Biofeedback

Biofeedback teaches self-regulation of autonomic functions related to stress and pain, including heart rate, respiratory rate, and skin temperature. A meta-analysis, including five studies with 137 participants, found that biofeedback reduced pediatric migraine frequency (Mean Difference (MD) −1.97 (95% CI, −2.72–−1.21); *p* < 0.00001), attack duration (MD −3.94 (95% CI, −5.57–−2.31); *p* < 0.00001), and headache intensity (MD, −1.77 (95% CI, −2.42–−1.11); *p* < 0.00001) compared with waiting-list control patients [11].

The CMH biofeedback clinic is staffed by a clinical pain psychologist and trained nursing staff. The biofeedback approach utilizes audio-visual technology to teach regulation of heart rate, respiratory rate, and skin temperature. Additionally, the pain psychologist utilizes cognitive behavioral therapy techniques, including acceptance and commitment therapy, to facilitate generalization and application of strategies in pain management. Patients who consistently practice self-regulation skills independently see the most success with this program. 

#### 2.4.2. Relaxation Training

Relaxation training includes deep/diaphragmatic breathing, progressive muscle relaxation, guided imagery, and mindfulness/meditation. Relaxation training, like biofeedback, increases patients’ control over physiological responses to headache pain, reducing stress and anxiety, and decreasing sympathetic overdrive. While little data exists on relaxation in children, progressive muscle relaxation (PMR) was shown to decrease migraine frequency in adults by up to 41% after a six-week intervention [12]. In a meta-analysis, relaxation training in adult studies demonstrated an effect size of 0.55 (CI: 0.14–0.96), representing a moderate improvement in migraine headaches [28].

The CMH headache providers utilize a website, www.headachereliefguide.com, to teach relaxation strategies to patients and families. The website, developed by Dr. Jennifer Bickel, MD and Dr. Mark Connelly, PhD, contains a “relaxation section” with videos demonstrating relaxed breathing, PMR, and guided imagery. Additionally, CMH pain psychologists teach relaxation strategies during some headache clinic visits. 

#### 2.4.3. Hypnosis

Clinical hypnosis is a self-regulation strategy using self-directed suggestions to facilitate the mind-body connection, ultimately cultivating a sense of awareness and positive well-being [29]. A clinical hypnosis case series demonstrated a therapeutic benefit for adolescents with comorbid chronic daily headaches and anxiety [13]. Mind-body strategies, including clinical hypnosis, may regulate connections in the prefrontal structures of the adolescent brain, reducing the impact of stress-related conditions [30]. Mind-body therapies also balance dysregulation of the autonomic nervous system that may occur in migraines [31,32].

A headache-trained nurse practitioner at CMH received certification in clinical hypnosis at the National Pediatric Hypnosis Training Institute. This nurse practitioner offers clinical hypnosis during clinic visits to assist children and teens in self-guided relaxation, thus optimizing resilience.

#### 2.4.4. Psychological Services

Depression and anxiety may contribute to functional impairment in children and adolescents with migraines [33]. A 2016 meta-analysis found that cognitive behavioral therapy demonstrated clinically significant improvement (50% or greater reduction of headache activity) after treatment and at follow-up three months later (Odds Ratio (OR) 9.11 (95% CI: 5.01–16.58, *p* < 0.001) and OR 9.18 (95% CI: 5.69–14.81, *p* < 0.001), respectively) [14]. A 2014 Cochrane review demonstrated that face-to-face psychological treatments reduced pain intensity in pediatric headache patients, with long-term benefits [34].

CMH Headache Clinic patients who are missing multiple days of school due to headache and/or have comorbid psychological concerns are referred to the CMH Comprehensive Headache Clinic. In this multidisciplinary clinic, patients and their families are treated by a headache physician, a clinical pain psychologist, and a headache social worker. Pain psychologists provide education in relaxation and self-regulation, pain coping strategies, and lifestyle modification. Additionally, acceptance and commitment therapy and other cognitive behavioral strategies are utilized. Pain psychologists frequently help patients and their families to find counseling services near their homes, facilitating ongoing psychological care. The headache social worker provides parental support and headache-specific school accommodations. This often includes a school letter and, when indicated, a 504 plan. 

#### 2.4.5. Lifestyle Strategies

Nutrition, sleep, water, caffeine, and physical activity all affect headaches. Skipping meals, not getting enough sleep, and decreased physical activity have been associated with recurrent headaches in the adolescent population [35]. Modifying sleep hygiene in children may play a significant role in improving headache symptoms [36]. Caffeine and food additives including monosodium glutamate, cocoa, aspartame, cheese, citrus, and nitrites are common triggers for headaches in children [37]. Caffeine abstinence has been associated with improved headache outcomes [38]. Increasing water intake may result in increased quality of life and decreased headache duration [39,40]. 

CMH Headache Clinic practitioners evaluate and discuss lifestyle factors at each clinic visit, often helping patients to identify a particular lifestyle factor on which to focus their improvement efforts for the next few months. Headache Clinic nurses provide additional patient education at the end of clinic visits, and written information is also provided. Additionally, patients and families are referred to www.headachereliefguide.com to review lifestyle recommendations. Lifestyle “check-ins” are done through telephone triage by nursing staff. Parents are encouraged to call a live nurse phone line regarding questions, concerns, and updates regarding their child. Nursing staff routinely ask about lifestyle behaviors and provide education, utilizing provider recommendations and the headache relief guide website. This empowers patients and families to take ownership of their own health outcomes.

## 3. Case Studies

All names have been changed to protect privacy.

### 3.1. Case Study: Claire

Claire is an 18 year-old female with diagnoses of migraine without aura, vestibular dysfunction, and ulcerative colitis. At age 14, she sought care with a neurologist at an outside facility. At that time, headaches occurred once a week. Claire’s headaches worsened at the age of 15 after sustaining three mild traumatic brain injuries within a six-week time period. Screening labs and brain MRI were normal. Claire’s headaches were refractory to multiple medications including nortriptyline, propranolol, topiramate, amitriptyline, CoQ10, zolmitriptan, hydrocodone-acetaminophen, and two OnabotulinumtoxinA treatments. She had not attended school for one year (due to headaches) prior to her first appointment at the CMH Headache Clinic.

Claire began treatment with a nurse practitioner at the CMH Headache Clinic at the age of 16. At her initial visit, she reported continuous occipital headaches associated with visual scotoma, dizziness, and nausea. Her nurse practitioner noted signs of vestibular dysfunction on exam and referred Claire to vestibular therapy, in addition to initiating preventive and abortive medications. The nurse practitioner counseled Claire on the importance of good headache hygiene, specifically focusing on increasing water intake and regulating sleep. Claire was receiving counseling for stress, which her nurse practitioner recommended continuing. Claire received occipital nerve blocks twice after that visit without headache improvement. She tried several preventives including tizanidine, cyproheptadine, atenolol, and lisinopril. She practiced vestibular exercises consistently and the dizziness partially improved.

Claire’s nurse practitioner ordered the CMH comprehensive aggressive migraine protocol (CAMP) after nine additional months of intractable headaches despite multiple interventions. CAMP is a five-day outpatient dihydroergotamine (DHE) and magnesium infusion program. CAMP patients and their parents receive visits from a nurse practitioner, pain psychologist, and social worker. Massage, aromatherapy, hypnosis, Cefaly (an FDA-approved transcutaneous electrical nerve stimulation unit device to prevent and abort headaches), and acupuncture can also be provided during a CAMP session. Claire’s headache decreased from 6 out of 10 to 1 out of 10 on the VAS by the end of the fourth day of CAMP.

Claire began monthly acupuncture sessions after CAMP. Acupuncture procedures varied at each monthly session and included electroacupuncture, scalp, auricular, neck and back, abdominal, and extremity acupoints. After each acupuncture session, she had two to three weeks of complete headache relief. She was able to wean off of preventive medication completely for several months but has since restarted daily amitriptyline and occasionally requires rizatriptan or a combination abortive treatment of ibuprofen, an antiemetic, and an antihistamine. She now attends college and is managing classes and a part time job successfully. She continues to receive monthly acupuncture for prevention of migraines and sees her nurse practitioner at regular intervals.

### 3.2. Case Study: Allison

Allison is an 18 year-old female with diagnoses of chronic daily headache, asthma, allergic rhinitis, and anxiety. She began treatment with a nurse practitioner at the CMH Headache Clinic at the age of 16, approximately three months after sustaining her third concussion. Her examination showed vestibular dysfunction but was otherwise normal. Allison’s nurse practitioner ordered vestibular therapy, magnesium oxide daily for headache prevention, and an abortive cocktail of ibuprofen and diphenhydramine. Her abortive cocktail was limited to two to three times weekly. Lifestyle counseling on hydration, nutrition, sleep, and stress was given at the time of visit and www.headachereliefguide.com was used to teach relaxation strategies.

At her three-month follow-up visit, Allison had been compliant with her medication regimen, vestibular therapy, and lifestyle modifications. She continued to have daily headaches, but with decreased headache intensity. Additionally, she reported dizziness, fogginess, and increased anxiety. Her headache provider added a second preventive medication, amitriptyline daily, and encouraged her to initiate counseling. She was prescribed a new abortive cocktail of hydroxyzine, prochlorperazine, and ibuprofen. She was also referred for biofeedback therapy.

Allison started weekly biofeedback visits with a trained psychologist for a total of five weekly sessions. Biofeedback entailed utilizing four different sensors that measure physiologic correlates to stress or autonomic regulation: A surface electromyograph on the frontalis muscle of the forehead to measure muscle tension, a temperature sensor on pad of middle finger for thermoregulation and vascular reactivity, an electrodermal sensor on index and fourth fingers to measure skin conductance (i.e., sweat/moisture level), and a pneumograph strain gauge just below the rib cage to measure respiratory patterns. At the first visit, Allison exhibited sympathetic and vascular activation in response to stress. She was instructed to practice diaphragmatic breathing for three minutes, three times daily. At the second session, she was taught PMR. By the third session, her daily headaches had decreased to one headache per week. She completed a guided imagery exercise and was instructed to practice this in addition to diaphragmatic breathing and PMR at home. At her fourth session, she reported one headache per week, which responded well to abortive therapy. She was also taught autogenic relaxation during her fourth biofeedback session. At her fifth session, she continued to only report one headache weekly. At the end of biofeedback, she demonstrated improvement in all four biofeedback measures including reduced heart rate, reduced muscle tension on electromyography, increased skin temperature, and reduced respiratory rate. This is indicative of improvements in her ability to regulate stress or the autonomic nervous system. After biofeedback, she has continued practicing autogenic relaxation, guided imagery, PMR, and diaphragmatic breathing exercises at home.

Three months following biofeedback treatments, Allison returned for a follow-up Headache Clinic visit. Her exam, including vestibular function, was normal. Allison’s headaches were still transformed from chronic daily headaches to episodic as she reported only one headache per week, alleviated by ibuprofen and sleep. She was advised to continue lifestyle recommendations and biofeedback techniques to help manage and prevent headaches, along with daily amitriptyline for prevention and an abortive cocktail as needed.

### 3.3. Case Study: Makayla

Makayla is a 13 year-old female with diagnoses of migraine headache, ankle pain, asthma, chronic urticaria, food allergies, and vasomotor rhinitis. She began treatment with a Headache Clinic nurse practitioner for post-concussion syndrome, after sustaining several concussions. She reported that her migraine headaches began at age seven, with worsening of headache frequency and severity after her concussions. Her post-concussive symptoms included daily continuous headaches and severe migraines three to four days per week, accompanied by light, noise, and smell sensitivity, nausea, numbness, weakness, tingling, vestibular dysfunction, daytime sleepiness, fatigue, and allodynia. She did report that school had become a major stressor for her with difficulty concentrating and not being able to keep up with the work. However, she denied suicidal ideation or poor mood. At the initial visit, she had missed 15 days of school in one semester and had discontinued sports participation after her concussions. She was referred for vestibular therapy and counseled in lifestyle factors, including nutrition, sleep, hydration, and stress management. Prior to her initial headache visit, Makayla was utilizing ibuprofen or acetaminophen on an as needed basis without relief. Her nurse practitioner initiated tizanidine and magnesium gluconate daily for headache prevention. Additionally, meloxicam daily was initiated for 30 days, and rizatriptan was prescribed as a headache abortive, limited to three times weekly. 

Additional therapies included Cefaly, an occipital nerve block, and acupuncture. Cefaly was trialed at her clinic visit, with a subsequent decrease in VAS pain scores from 4 out of 10 to 3 out of 10. She trialed Cefaly during a later clinic visit, with similar results. When Makayla underwent an occipital nerve block with lidocaine and bupivacaine, she developed a delayed hypersensitivity reaction to lidocaine with worsening headaches after the nerve block. She was then prescribed prochlorperazine, diphenhydramine, and a solumedrol dosepak for the severe headache. However, these medications did not result in headache relief. Acupuncture was initiated to include scalp, auricular and extremity points, with six acupuncture sessions over eight weeks. At her first acupuncture visit, her headache decreased from 7 out of 10 to 4 out of 10 on the VAS. Additionally, she received counseling in relaxation, acupressure, and diaphragmatic breathing. The following day, she received intramuscular DHE, metoclopramide, ondansetron, and diphenhydramine along with acupuncture. Her pain decreased from 4 out of 10 to 2 out of 10 on the VAS. On subsequent acupuncture visits, she presented with 3 to 4 out of 10 headache pain, decreasing to 1 to 2 out of 10 on the VAS after acupuncture.

Makayla was also referred for clinical hypnosis with a nurse practitioner trained in pediatric clinic hypnosis. Makayla participated in several 40 min hypnosis sessions that focused on progressive muscle relaxation and imagery of a favorite place. The favorite place was a place known only to Makayla where she felt safe and calm. At the end of the visit she was taught self-hypnosis and self-relaxation techniques to trial at home, and she practiced this twice daily for 30 min. Makayla also began counseling sessions with a psychologist. At follow-up visits, she has reported that her headaches are mild and infrequent, occurring less than four times monthly.

## 4. Conclusions

Integrative therapies offer non-pharmacological treatment options that effectively reduce headache frequency and severity. The goal of integrative therapies is to empower individuals to take ownership of their health through a holistic patient-provider relationship that emphasizes education and at-home adherence. The CMH Headache Clinic has demonstrated positive outcomes by incorporating integrative therapies into headache treatment plans. Headache treatment in the clinic is as multifaceted as the headaches themselves. The multidisciplinary approach takes this complex etiology into account by addressing factors such as lifestyle, anxiety, and stress, through a variety of services.

Concerns about financing integrative therapies are relevant, as integrative therapies may not be covered by government or commercial insurance. CMH takes part in both government and commercial insurance for reimbursement of integrative services, such as acupuncture, biofeedback and relaxation, and psychology services. While financial sustainability is a main priority for healthcare organizations, incorporating evidenced-based treatments that improve clinical outcomes and quality measures should be prioritized. 

Furthermore, future studies on integrative therapies and headaches should include larger sample sizes and randomized controlled trials specific to pediatric patients.
